# The association of acute alcohol use and dynamic suicide risk with variation in onward care after psychiatric crisis

**DOI:** 10.1111/dar.13231

**Published:** 2021-02-10

**Authors:** John E. Robins, Nicola J. Kalk, Kezia R. Ross, Megan Pritchard, Vivienne Curtis, Katherine I. Morley

**Affiliations:** ^1^ National Addiction Centre King's College London Institute of Psychiatry, Psychology and Neuroscience London UK; ^2^ South London and Maudsley NHS Foundation Trust London UK; ^3^ NIHR Maudsley Biomedical Research Centre London UK; ^4^ King's College London Institute of Psychiatry, Psychology and Neuroscience London UK; ^5^ School of Psychiatry, Health Education England London UK; ^6^ Innovation, Health, and Science, RAND Europe Cambridge UK; ^7^ Centre for Epidemiology and Biostatistics Melbourne School of Global and Population Health, The University of Melbourne Melbourne Australia

**Keywords:** psychiatric‐emergency‐services, suicide, electronic‐health‐records, alcohol drinking, patient‐discharge

## Abstract

**Introduction:**

Despite the association of alcohol use with recurrent suicidal acts, individuals attempting suicide after drinking alcohol face barriers accessing crisis care following emergency assessment, demonstrated by higher odds of inpatient admission for those whose suicide attempt did not feature alcohol. This disparity may be due to suicidality dissipating more rapidly after a suicide attempt involving alcohol. We investigated the effect of acute alcohol use and ongoing suicidality on onward care decisions after emergency assessment.

**Methods:**

We analysed electronic health records of 650 suicidal adults detained under Section 136 of the *Mental Health Act* (1983, amended 2007) for up to 36 h at a London psychiatric emergency care centre. We used logistic regression to estimate the association of acute alcohol use and ongoing suicidality (including their interaction) with admission to psychiatric hospital.

**Results:**

Fifteen percent of previously intoxicated detainees expressed suicidal intent at detention end, compared to 24% of detainees who had not used alcohol prior to detention. Compared to those who were not previously intoxicated and not suicidal at detention end, acute alcohol use was associated with reduced odds of admission amongst those no longer suicidal (AOR 0.4, 95% CI 0.2, 0.6). Where suicidality persisted, odds of admission rose; however, the magnitude of increase when in combination with prior alcohol use (AOR 3.6, 95% CI 1.9, 7.1) was under half that of when alcohol was not involved (AOR 8.2, 95% CI 3.5, 19.1).

**Discussion and Conclusions:**

Acute alcohol use is associated with transient suicidality, but this only partially accounts for disparities in care following suicide attempts.

## Introduction

Suicide is the second leading cause of death worldwide for people aged 15–29 years [1]. One in five suicides is attributable to alcohol [1, 2, 3]. Although acute alcohol use is associated with repeated self‐harm and completed suicide [4, 5], alcohol users are more likely to be discharged home after emergency care following a suicide attempt than the non‐intoxicated [6]. While there are many potential explanations, data are limited on how this relates to the dynamic nature of both alcohol intoxication and suicidal ideation. The anecdotal observation reported by clinical colleagues is that as people sober up, their suicidal ideation tends to subside, along with the necessity for treatment or acute containment of risk [7]. Few studies have explored how suicidality changes following a suicide attempt. Smartphone‐enabled Ecological Momentary Assessment demonstrated that suicide risk can fluctuate throughout a day [8], and 44% of those hospitalised in the USA due to suicidality no longer reported suicidal ideation 24‐h later [9]. However, the role of alcohol intoxication was not considered. We therefore aimed to understand whether suicidality resolved in a greater proportion of people who had used alcohol in the setting of a suicide attempt and whether this accounted for differences in onward care.

**Figure 1:**
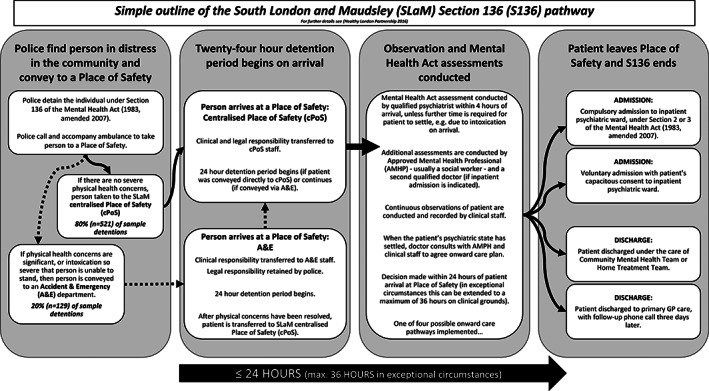
Care pathway for people detained under section 136 of the *Mental Health Act* (1983, amended 2007).

Conducting research on individuals in suicidal crisis can be challenging [10], but studying individuals in emergency care settings can help to illuminate the relationship between alcohol use and suicidal acts [11]. In England and Wales, police can use Section 136 (s136) of the *Mental Health Act* (MHA) (1983, amended 2007) to detain people in psychiatric crisis who are ‘in immediate need of care or control’ and take them to a Place of Safety for emergency psychiatric assessment [12, p. 104]. A place of safety denotes a dedicated site where an MHA assessment can be conducted in a non‐punitive environment, by a team trained in a range of physical and mental health crisis care competencies [13, 14]. The duration of detention is a maximum of 36 h (see Figure 1), and the majority of individuals detained are intoxicated with alcohol and/or other drugs [15]. Following assessment, individuals are either admitted to a psychiatric ward voluntarily or compulsorily for further assessment and treatment, or discharged to secondary community mental health services or primary care.

We used pseudonymised electronic health record data from over 800 s136 detentions to examine the interaction between acute alcohol use with change in suicidal intent during psychiatric crisis, and the extent to which this is associated with variation in onward care. Our focus was the characteristics of individuals immediately prior to and during the period of detention, as our aim was to investigate how these acute factors contribute to clinical decision‐making. We focussed on alcohol use because previous research in this setting and in emergency departments indicates that alcohol use is overwhelmingly the most common substance used in association with suicidal crisis [15, 16]. Previous research indicates that acute alcohol use increases risk of suicide irrespective of alcohol use disorder [17]. Our hypothesis was that acute alcohol use and active suicidal thoughts interact, such that suicidal individuals who have used alcohol prior to detention are less likely to report active suicidal thoughts at the point where decisions about onward care are made, and will be less likely to be admitted to a psychiatric ward and more likely to be discharged to community follow‐up.

## Methods

### Sample

We analysed data from individuals detained at the South London and Maudsley NHS Foundation Trust (SLaM) centralised Place of Safety (cPoS) under s136 of the MHA [12]. All detentions of individuals aged 18 years and over between 1 February 2017 and 4 October 2018 were eligible for inclusion. The SLaM cPoS also receives individuals detained under Section 135 of the MHA, whereby a magistrate has approved the removal of a person from their home to facilitate an MHA assessment (see Figure S1, Supporting Information) [12]. Such detentions accounted for under 10% of the sample and represent a distinct group where MHA assessment is planned, and were thus excluded. Similarly, detentions of those diagnosed with dementia or learning disability recorded during detention or in the year prior were excluded, as these individuals are rarely detained in the cPoS and have distinct needs and care pathways. A minority of individuals were detained multiple times during the study period; primary analysis was restricted to the first detention of each individual to prevent bias via repeat measurement.

Data were drawn from the pseudonymised electronic health record (EHR) collection of SLaM patient records available via the SLaM Biomedical Research Centre Clinical Records Interactive Search system (CRIS) [18]. We used information from structured fields derived from structured forms and unstructured text fields completed by clinicians.

Information from unstructured notes was extracted using natural language processing algorithms and manual review. Previously created natural language processing algorithms were used to extract information on diagnoses and prescribed medications [18]. Manual review of unstructured text fields was used to extract sociodemographic information, psychiatric symptoms and substance use. All patient identifiable information was removed prior to use by the CRIS application, including patient, family or friends' names, and location information. All data remained within the NHS firewall during analysis. Text was reviewed by investigators and data entered into a database using a custom data entry interface. A subset of unstructured text was re‐scored by NJK, a consultant psychiatrist, to estimate inter‐rater reliability; estimates of Cohen's kappa differed by variable but were generally strong, ranging from 0.72 to 0.94. Database queries and data extraction, processing, entry and analysis were all implemented in R software (version 3.5.1) [19].

All procedures contributing to this work comply with the Helsinki Declaration of 1975, as revised in 2008. Ethical approval was granted via the Oxford C Research Ethics Committee (NRES: 08/H0606/71 + 5), which covers use of CRIS as an anonymised database for secondary analysis [18]. Approval for this project was obtained from the CRIS oversight committee (17–104). Written or verbal consent from patients was not required as the data used were pseudonymised, however, use of these data for research via CRIS is publicised across the SLaM NHS Trust, including the option for service users to opt out.

### Measures

#### Initial suicidality

There are currently no structured fields used in cPoS which capture presenting circumstances prior to s136 detainment, but this information is routinely recorded in the unstructured text fields. Unstructured fields were screened to determine the presence or absence of suicidal thoughts and/or suicidal acts precipitating detention and method(s) used. We combined suicidal thoughts and acts because for people detained under s136, expressions of suicidal intent were deemed to warrant ‘immediate care or control’ [12, p. 104] by police and were therefore considered credible threats, and the distinction between intent and actions is partially influenced by how quickly police responded. Initial suicidality was scored as ‘Yes’ if police or clinical staff reported suicidal thoughts or acts precipitating detention; ‘Possible’ if the patient's reported actions could be interpreted as suicidal—such as walking into traffic—but intent was not clearly present; and ‘No’ if no suicidal thoughts or acts were reported. For analysis purposes, only service users categorised ‘Yes’ were included in the sample.

#### Acute alcohol use

Detentions were categorised as involving acute alcohol use if any of the following criteria were met:

- Blood alcohol content above 0.00%;
- Clinical Institute Withdrawal Assessment for Alcohol Scale score above 0 [20];
- Alcohol detoxification medication (chlordiazepoxide) provided at cPoS;
- Structured EHR field indicating alcohol intoxication set to ‘Yes’ (see Supporting Information);
- Recorded on the Current Drug and Alcohol Assessment (part of standard cPoS assessment) or in unstructured fields as having used alcohol on day of detention.

We developed hierarchical algorithms for integrating this information to determine whether there was evidence for acute use of alcohol, which was categorised as a binary indicator (see Supporting Information).

#### Ongoing suicidality

As there are currently no structured fields in CRIS to capture changes in suicidal intent during cPoS detention, this information was extracted from review of unstructured text fields. Assessment of suicidality is a significant component of risk assessment which is important in making decisions about onward care, particularly discharge, and is thus reliably documented. Classification of individuals as having persistent suicidality was drawn from text authored by senior doctors at the time where onward care decisions were made. This was scored as ‘Yes’ if the patient could not guarantee their safety and/or continued to report specific intent or plans to end their life; ‘Possible’ if they reported ambivalence about their survival but no specific suicidal intent or plans, and ‘No’ if they reported they no longer wanted to die and demonstrated future orientation. For analysis purposes ‘No’ and ‘Possible’ were combined due to the small number of individuals in the latter category.

#### Detention outcome

Detention outcome was determined from several different structured data sources (see Supporting Information). For analysis, we collapsed these categories to a binary variable; ‘Admission’ encompassed voluntary or compulsory admission and ‘Discharged’ encompassed Home Treatment Team, Community Mental Health Team or GP follow‐up.

#### Recent substance use

Data on recent substance use were collated in a similar way to acute alcohol use, including the results of urine drug screens. We developed hierarchical algorithms for integrating this information to determine whether there was evidence for recent use of each substance of interest (see Supporting Information), which were aggregated to create a binary indicator of recent use of any non‐prescribed drugs.

#### Diagnoses

Psychiatric diagnoses recorded during the past year (up to and including the current detention) were drawn from the structured diagnosis field and from the output of a previously developed natural language processing algorithm [18]. Psychiatric diagnoses were categorised as ‘Psychotic’ or ‘Non‐psychotic’ with an additional category of ‘None’ where no psychiatric diagnoses had been recorded (codes for substance use disorders and physical health diagnoses were excluded; see full list of International Classification of Diseases, 10th Revision codes and categorisation in Table S3). We used these three categories as service users with psychotic diagnoses have greater odds of psychiatric admission following emergency assessment in settings similar to the current study [21]; which is also observed in international data on involuntary psychiatric hospital admissions [22]. Where multiple diagnoses existed that belonged to different categories, psychotic illness diagnoses were preferentially retained.

#### Sociodemographic

Age in years at date of detention was treated as a continuous variable. Sex was a binary variable, female or male. Ethnicity was a binary variable indicating ‘White’ and ‘Non‐white’ collapsed from the range of structured field options in CRIS. Housing was a binary variable derived from structured and unstructured fields (‘Stable’ or ‘Unstable’; see Supporting Information).

### Statistical analysis

Descriptive analyses were conducted using means, standard deviations, counts and proportions as appropriate for variable type. Bivariable associations with the primary outcome variable, detention outcome, were conducted using *t*‐tests, χ^2^ tests or Fisher's exact test as appropriate. For the primary analysis, multivariable binomial logistic regression was used to model the association between detention outcome and the interaction between acute alcohol use and ongoing suicidality. We adjusted for age, sex, ethnicity, housing status, psychiatric diagnoses and recent other drug use. As this study is explanatory rather than predictive, all variables were included in the model and no further selection strategies were implemented [23]. Results for the interaction term are presented following published guidance [24, 25]. Whether an interaction is present, and the effect it has, is determined by whether an additive or multiplicative approach is used to model it. An additive interaction occurs when the combined effect of two risk factors is larger or smaller than their individual effects added together; for a multiplicative interaction this combined effect is larger or smaller than the multiplication of the individual effects [26]. Interactions from logistic regression models are usually presented on the multiplicative scale, although the additive scale is generally viewed as more appropriate for interactions of public health or biological importance [24]. Consequently, we estimate the significance of the interaction on both the multiplicative and additive scales, the latter estimated using the Relative Excess Risk due to Interaction measure, interpreted as the risk additional to that expected based on the simple addition of the two exposure risks [26]. For transparency, the full model results are also presented as regression coefficients with 95% confidence intervals (CI).

We imputed missing covariate values using Multiple Imputation by Chained Equations as implemented for the R software [27], following guidance for implementation and reporting (see Supporting Information) [28, 29]. We initially investigated variable‐wise and participant‐wise missing data, and the distribution of variables in complete cases versus those with missing data. As recommended, the multiple imputation model included the primary and secondary outcomes, all predictors, accounting for pre‐planned interactions and relevant auxiliary variables [30, 31, 32, 33]. Fifty imputed data sets were generate*d. Variable* distributions from observed and imputed data sets were compared, and results from analyses of the individual datasets combined using Rubin's rules [34, 35].

We conducted two types of sensitivity analysis to investigate the robustness of our results. First, we assessed the impact of departures from the Missing at Random assumption on results [36]. Second, we examined the impact of analysing data at the *detention*‐level rather than the *individual*‐level, by including the previously excluded repeat‐detentions of those service users detained more than once in the study period. Further details are provided in Supporting Information. All tests were two‐tailed but thresholds for declaring statistical significance were not applied; effect estimates, confidence intervals and *P*‐values were evaluated together to determine the importance of results [37, 38].

## Results

### Sample description

**Figure 2:**
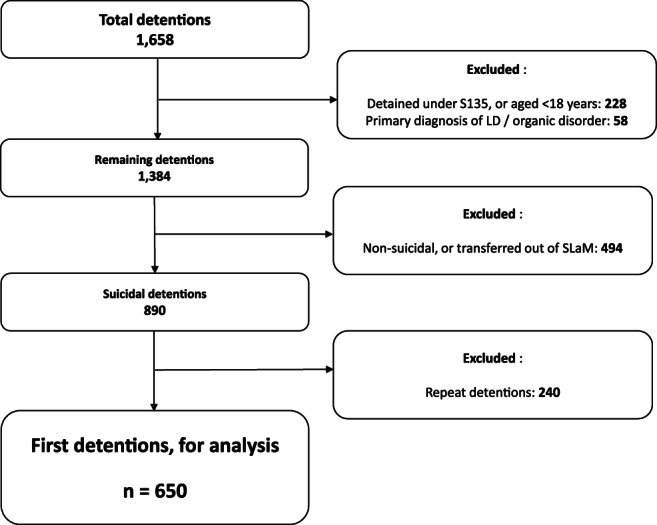
Application of exclusion criteria to initial dataset. S135 detentions indicates detention under section 135 of the *Mental Health Act* (1983, amended 2007); LD indicates learning disability diagnosis. Some exclusion criteria combined to avoid reporting counts <10. SLaM, South London and Maudsley NHS Foundation Trust.

During the study period, there were 1658 detentions to the cPoS under the MHA. After applying inclusion/exclusion criteria (see Figure 2), the sample consisted of 890 detentions related to suicidal acts or threats. Of these, 650 represented the first detention of an individual during the study period, and these 650 were thus used as the main analysis sample. The sample contained more males than females (60%; *n* = 384; see Table 1) and mean age was 35 years (SD 12 years). In 60% of detentions, there was evidence of acute alcohol use prior to detention (*n* = 392). Suicidal intent and plans appeared to have resolved prior to decisions about onward destination in 82% of detentions (*n* = 531).

**Table 1:** Descriptive statistics based on first detentions only (n = 650) displayed as number and percentage, or mean and standard deviation

			Detention outcome				
			Discharge		Admission		
Variable	Range	Total detentions	M	SD	M	SD	*P*
Age, years	18–70	650	35	12	34	11	0.19

			Detention outcome				
			Discharge		Admission		
Variable	Categories	Total detentions	*n*	%	*n*	%	*P*
Acute alcohol use	Yes	392	316	69.1	76	39.4	<0.0001
	No	194	107	23.4	87	45.1	
	Missing	64	34	7.4	30	15.5	
Ongoing suicidality	No	531	417	91.2	114	59.1	<0.0001
	Yes	109	37	8.1	72	37.3	
	Missing	10	*	*	*	*	
Past‐year psychiatric diagnosis	Non‐psychotic	320	243	53.2	77	39.9	<0.0001
	Psychotic	156	73	16	83	43	
	None	174	141	30.9	33	17.1	
Sex	Female	266	188	41.1	78	40.4	0.93
	Male	384	269	58.9	115	59.6	
Ethnicity	White	419	314	68.7	105	54.4	<0.001
	Non‐white	166	94	20.6	72	37.2	
	Missing	65	49	10.7	16	8.3	
Housing status	Stable	465	337	73.8	128	66.3	0.03
	Unstable	138	86	18.8	52	27	
	Missing	47	34	7.4	13	6.7	
Other recent drug use^a^	No	336	245	53.6	91	47.2	0.72
	Yes	250	178	38.9	72	37.3	
	Missing	64	34	7.4	30	15.5	

* indicates exact numbers suppressed due to low cell counts. *P*‐values derived from *χ* ^2^ tests or *t*‐test as appropriate for variable type.

^a^Other recent drug use includes cannabis, amphetamines, cocaine, opiates, sedatives, synthetic cannabinoids, MDMA, G, GHB/GBH.

Most detentions (70%; *n* = 457) ended in discharge; of these, 47% were discharged to secondary mental health services, with the remainder to primary care. In unadjusted analyses, acute alcohol use, ongoing suicidality, past‐year psychiatric diagnosis, housing status and ethnicity were associated with detention outcome (Table 1). There was no association between detention outcome and sex, age or recent use of other drugs. Acute alcohol use was associated with change in suicidal intent ( *χ* ^2^ = 5.4, df = 1, *P* = 0.02). There were missing data for all variables except for age, sex and past‐year psychiatric diagnosis. The complete case data set consisted of 483 individuals (74% of the analysis sample). There was no significant association between presence of missing data and any of the variables.

### Alcohol use, suicidality and detention outcome

Although the interaction term between acute alcohol use and ongoing suicidality was not statistically significant on the additive or multiplicative scales (*P* = 0.26 and 0.7, respectively; see Tables 2 and 3), we retained it in the model as this was a pre‐specified model component and subgroups comparisons were statistically significant. Compared to service users who had not used alcohol prior to detention and were no longer expressing suicidal thoughts or intent at the end of detention, service users who *had not* used alcohol and *were* still suicidal had the greatest odds of admission [adjusted odds ratio (AOR) 8.2, 95% CI 3.5, 19.1 *P* < 0.001]. For service users who *had* used alcohol and were still suicidal, the odds of admission rose but to a much lesser degree (AOR 3.6, 95% CI 1.9, 7.1, *P* < 0.001). Service users who had used alcohol prior to detention were less likely to be admitted than those who had not regardless of ongoing suicidality; the odds of admission were over two‐fold lower for service users who had used alcohol compared to those who had not in the group who were no longer suicidal (AOR 0.4, 95% CI 0.3, 0.6, *P* < 0.001), and in the group who remained suicidal at detention end (AOR 0.4, 95% CI 0.3, 0.8, *P* = 0.003). There was no association between use of drugs other than alcohol and detention outcome (Table 3). The only other predictors of admission were having a diagnosis of psychotic illness, and non‐white ethnicity.

**Table 2:** Estimates for interaction between acute alcohol use and ongoing suicidality for admission following detention, based on the multiply imputed data (*n* = 650)

	Acute alcohol use prior to detention		Acute alcohol use within strata of suicidal intent
	No	Yes	
*Ongoing suicidality at detention end*			
No	1.0	0.4 (0.2,0.6); <0.001	0.4 (0.3,0.6); <0.001
Yes	8.2 (3.5,19.1); <0.001	3.6 (1.9,7.1); <0.001	0.4 (0.3,0.8); 0.003
Persistent suicidal intent within strata of alcohol use	8.2 (3.5,19.1); <0.001	10.3 (4.5,23.8); <0.001	

Estimates are shown as odds ratio (95% confidence interval); *P*‐value. Measure of interaction on additive scale: Relative excess risk due to interaction (95% confidence interval) = 11.3 (−8.1,30.7); *P* = 0.26. Measure of interaction on multiplicative scale: ratio of odds ratios (95% confidence interval) = 1.3 (0.43,3.7); *P* = 0.7. Adjusted for age, sex, ethnicity, housing status, psychiatric diagnoses and other acute drug use.

**Table 3:** Estimates for coefficients for all predictors in the model, for admission after detention, with 95% confidence intervals (CI) and *P*‐values, fitted to the multiply imputed data (*n* = 650)

Variable	Value	Regression coefficient	95% CI	*P*
Acute alcohol use	No	Ref.		
	Yes	−1.05	−1.5 to −0.6	0.0001
Suicidal at detention end	No	Ref.		
	Yes	2.1	1.2 to 3.0	<0.001
Alcohol × suicidal		0.23	−0.8 to 1.3	0.7
Psychiatric diagnosis	Non‐psychotic	Ref.		
	Psychotic	1.62	1.1 to 2.1	<0.001
	None	0.26	−0.28 to 0.81	0.3
Age		−0.02	−0.03 to 0.56	0.5
Sex	Female	Ref.		
	Male	0.13	−0.3 to 0.6	0.6
Ethnicity	White	Ref.		
	Non‐white	0.47	0.01 to 0.9	0.05
Housing	Stable	Ref.		
	Unstable	0.36	−0.13 to 0.86	0.15
Other drug use^a^	No	Ref.		
	Yes	−0.03	−0.49 to 0.43	0.9

^a^Other recent drug use includes cannabis, amphetamines, cocaine, opiates, sedatives, synthetic cannabinoids, MDMA, G, GHB/GBH.

Results from the complete‐case analysis did not differ substantially, and the sensitivity analysis expanding the data set to include any repeat detentions within the study period produced similar results to the primary analysis (Supporting Information).

## Discussion

We found that acute alcohol use is associated with change in suicidality in those detained for emergency psychiatric care following a suicide attempt; 85% of those who had used alcohol prior to detention were not suicidal by the end of detention, compared to 76% in those who had not. However, this difference does not fully account for differences in onward care in those who use alcohol. Regardless of whether suicidality was ongoing or had resolved, where there was evidence of acute alcohol use prior to detention, individuals had approximately two‐fold lower odds of admission to inpatient psychiatric care compared to their non‐alcohol using counterparts.

Previous studies have speculated that disparities in onward care for those who used alcohol prior to a suicide attempt may be due to underreporting of suicidal ideation among the intoxicated, or unwillingness on the part of clinicians to assess such thoughts [39]. Our study suggests that these speculations are likely unfounded in this instance, as the discrepancy persisted among those still actively reporting suicidal intent. Also of note is the contrasting lack of association found between recent use of other drugs and detention outcome. This accords with previous research demonstrating an association of impulsive suicide attempts with alcohol use disorder but not drug use disorder [40], and underscores the importance of separating substances of abuse in research design. Our study further evidences the association of alcohol use with reduced odds of admission, as observed in other s136 samples [15, 41]. The increased odds of admission for those of non‐white ethnicity reflect international trends in MHA outcomes, for which multiple explanations are proposed and further research is warranted [42].

This study raises the question of what drives this difference in onward care beyond rapid reduction in suicidality. Those who attempt suicide in the context of alcohol use are heterogeneous in terms of alcohol use‐pattern and psychiatric comorbidity, and not all will benefit equally from hospital admission. Emotionally Unstable Personality Disorder—a diagnosis which is highly comorbid with alcohol dependence [43], and for which national guidelines discourage inpatient care [44]—may be prevalent in those who are discharged in our study, and this should be explored further.

Structural stigma may also play a role in that the needs of those attempting suicide following alcohol use may not be met by current treatment pathways. Previous research suggests that clinicians may view alcohol‐related suicide attempters as being less amenable to the treatment offered on inpatient wards. Consequently, they may be more likely to discharge, recommending engagement with secondary addiction treatment services such as supervised community detoxification and structured day‐programs, as endorsed by policy [45]. However, secondary addiction services may not accept or meet the needs of those who are not dependent but present suicide risk in response to a binge drinking pattern, or who may not view themselves as having an alcohol problem [7].

Stigma may be a barrier to accessing addiction and mental health services [46], as substance users are reported to be subject to stigmatising attitudes from clinicians [47]. As this was an EHR study and did not involve re‐contact of service users, it was not possible to conclude whether the onward care decisions reviewed here were inappropriate.

### Strengths and limitations

The strength of this study lies in its use of detailed clinical information from a difficult‐to‐study group, with no sampling bias. The sample of 650 individuals encompassed the first detention of all adults in suicidal crisis detained under s136 over a 20‐month period, within the NHS Trust serving the largest proportion of the UK population [18]. The only exceptions are those who would have remained in the accident and emergency department for physical health assessment or intoxication leading to incapacitation [14], or those with a primary diagnosis of dementia or learning difficulty.

Furthermore, recall bias is mitigated by the use of contemporaneously recorded EHR data. Nonetheless, EHR data is not entirely objective because clinicians summarise and prioritise certain information, and knowledge of what happened prior to contact with the health‐care system is dependent on information provided to clinicians by police, ambulance staff and the detained individuals themselves. Despite these limitations, this study demonstrates that disparities in onward care for suicidal individuals who have used alcohol are not wholly attributable to rapid resolution of suicidality.

In conclusion, our study highlights the need for interventions that account for the dynamic nature of suicide risk that acute alcohol use confers, but also the need for further research to explore other factors accounting for disparities in onward care that alcohol users face. This will include how different patterns of alcohol use cluster with psychiatric comorbidities and social factors, and how this influences the decisions that clinicians need to make in response to a potentially volatile level of risk.

## Conflict of Interest

The authors have no conflicts of interest.

## Supporting information


**Appendix** **S1.** Supporting Information.

## Data Availability

A pre‐registration for this study is available (DOI 10.17605/OSF.IO/KH87V). All authors had access to the study data during the period of data analysis. The ethical approval to access CRIS data (Oxfordshire Research Ethics Committee C 08/H0606/71 + 5) requires the data to be stored behind an NHS firewall with access governed by a patient‐led oversight committee. For this reason, the data cannot be made available in the manuscript, Supporting Information files or a public repository. However, subject to approval from the oversight committee, data access for research purposes is encouraged. Further information is available from cris.administrator@slam.nhs.uk.
