# Supplementary material for: The association of acute alcohol use and dynamic suicide risk with variation in onward care after psychiatric crisis
Source: Drug Alcohol Rev. 2021 Feb 10;40(3):499–508. doi: 10.1111/dar.13231 (PMC8647926; doi:10.1111/dar.13231)
Supplement: Supplementary file 1 — Appendix S1. Supporting Information. [file DAR-40-499-s001.docx]

The association of acute alcohol use and dynamic suicide risk with variation in onward care after psychiatric crisis

Supporting Information

John E. Robins, Nicola J. Kalk, Kezia R. Ross,
Megan Pritchard, Vivienne Curtis, Katherine I. Morley

**Contents**

[1 Clinical Pathways 2](#_Toc57842555)

[2 Phenotype Algorithms 2](#_Toc57842556)

[2.1 Background 2](#_Toc57842557)

[2.2 Data sources 2](#_Toc57842558)

[2.3 Algorithms 5](#_Toc57842559)

[2.3.1 Acute alcohol use 5](#_Toc57842560)

[2.3.2 Recent illicit and licit drug use 6](#_Toc57842561)

[2.3.3 Detention outcome and destination 7](#_Toc57842562)

[2.3.4 Past year psychiatric diagnoses 7](#_Toc57842563)

[3 Multiple Imputation 10](#_Toc57842564)

[3.1 Approach 10](#_Toc57842565)

[3.2 Results 11](#_Toc57842566)

[4 Sensitivity Analyses 13](#_Toc57842567)

[4.1 Approach 13](#_Toc57842568)

[4.2 Results 13](#_Toc57842569)

[References 15](#_Toc57842570)

# Clinical Pathways

As discussed in the main text, individuals could be detained to the Place of Safety under Section 135 or Section 136 of the *Mental Health Act* (1983, amended 2007). This study was focused on people detained under Section 136 and this pathway is discussed in more detail in the main text. We provide an equivalent diagram of the Section 135 pathways here for completeness (Figure [S1).](#_bookmark4)

# Phenotype Algorithms

## Background

The CRIS database is complex as the patient journey system is used by many different clinical services with different medical records needs. Consequently, information relevant to the definition of a single variable may be recorded in multiple database tables and free-text documents. We developed phenotype algorithms to combine data from these sources in the most efficient way, using a staged approach to variable definition:

- - 1. Identify sources of structured information: Data sources were identified via discussions with clinical and informatics colleagues, and examination of database table structure.
    2. Examine content of each structured information source: We examined how frequently the relevant database fields were populated with non-null values.
    3. Consider natural language processing (NLP) data sources: If relevant NLP algorithms for use with CRIS data already existed, we used these to extract information from clinical notes within the time period.
    4. Consider manual note scoring: If information was only available in clinical notes and could not easily be captured via NLP algorithms (e.g. suicide risk), we considered whether it could be converted into structured data via manual note scoring.
    5. Integrate structured and unstructured data: For those variables where data were extracted from more than one source, electronic health record phenotype algorithms were developed to define the strategy used to integrate and reconcile these data.

## Data sources

We included data from a number of different sources in patient journey system. Although the cPoS has its own structured proforma for collecting information, this was not implemented until approximately six months after the cPoS opened. Thus we had to combine information from this proforma with other data sources in order to extract the variables required for all detentions in the data set. A brief description of the different data sources, including whether we used structured or unstructured information from each source, is provided in Table [S1.](#_bookmark5)

3


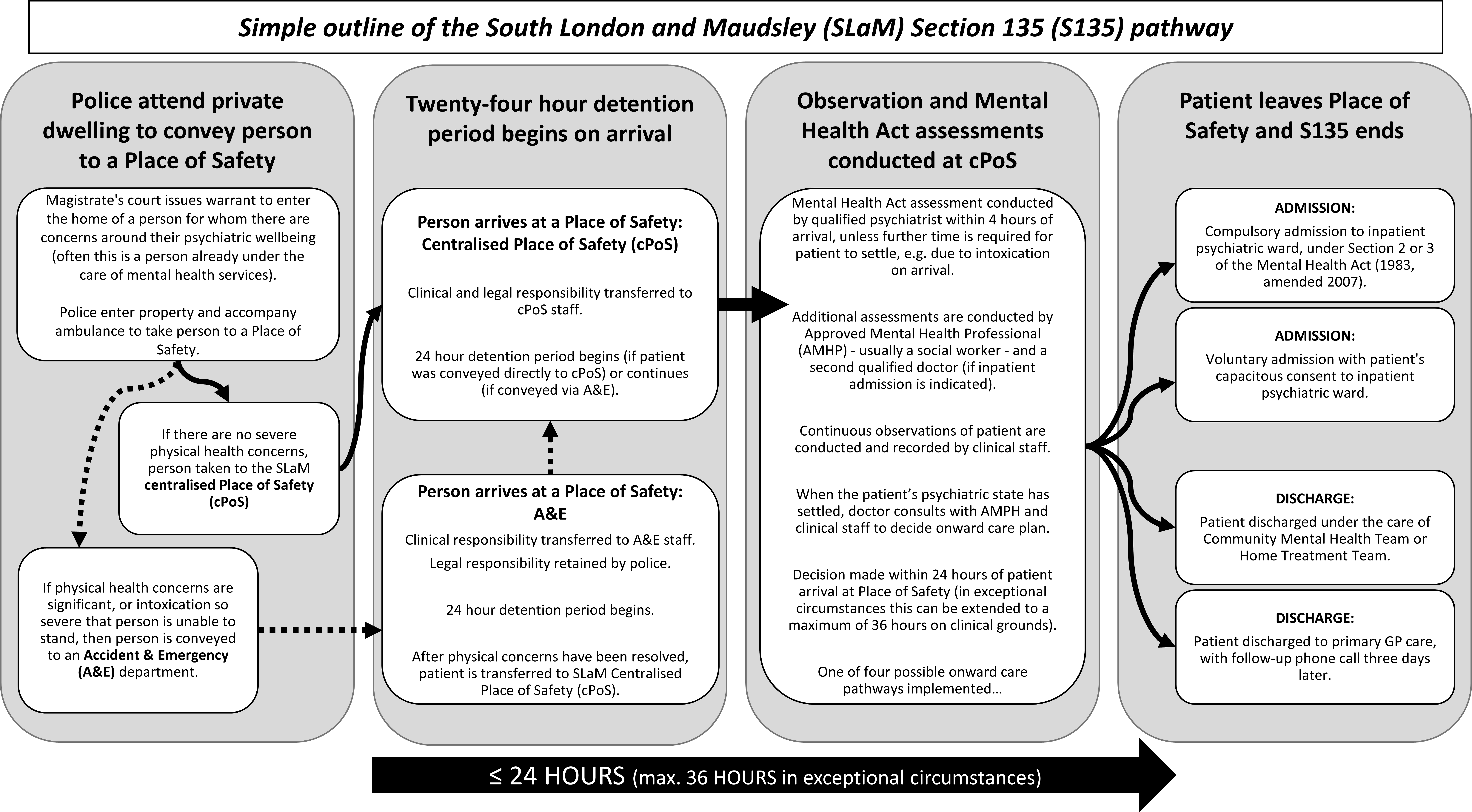


**Figure S1.** Diagram of pathway for detentions under Section 135 of the *Mental Health Act* (1983, amended 2007).

**Table S1.** Included CRIS data sources. Description of CRIS data sources from which data were extracted. Whether the data source contained structured (S) and/or unstructured (U) information used in generating phenotype algorithms is indicated.

| **Source** | **Description** | **Data Types** | |
| --- | --- | --- | --- |
|  |  | S | U |
| Electronic patient record | Records core patient sociodemographic information. | ✓ |  |
| Place of Safety Proforma | Custom form designed to record large range of information relating to detention characteristics including mode of transportation, use of restrictive interventions by police, alcohol and drug intoxication, detention outcome and destination, clinical notes from all health and social care professionals relating to detention. | ✓ | ✓ |
| Diagnosis | Can be used to record up to 6 ICD-10 diagnoses and allows Multi-Axial ICD-10 recording. Diagnoses are only ‘registered’ within NHS Trust reporting and performance systems via this form. A diagnosis can be assigned at any point, but is formally required: (i) following a mental state assessment and formulation; (ii) at inpatient discharge; (iii) at transfer/discharge from a community-based team. | ✓ |  |
| Current Drug And Alcohol form | Record of patient’s current drug and alcohol use. Includes specific fields for substance use on day of assessment, and on the day prior to assessment. | ✓ |  |
| Urine drug screen | Records urine screen results, which includes tests for a selection of prescribed medications and illicit drugs (cannabis, stimulants, opiates, sedatives). | ✓ |  |
| Alcohol Use Disorders Identification Test | Records the results of the Alcohol Use Disorders Identification Test | ✓ |  |
| Risk sssessment | Risk assessment should be recorded for all new service-users and updated whenever there is a significant change to the risk profile or at key intervals during the patient journey, such as at points of transfer between teams. Covers: risk to others (e.g. violence, sexual offending, stalking); risk to self (e.g. deliberate or accidental self- harm, self-neglect); risk from others (abuse, neglect, or exploitation). | ✓ | ✓ |
| Events | Notes for all clinical events |  | ✓ |
| *Mental Health Act* | Records all information about use of the Mental Health Act in clinical care | ✓ |  |

ICD-10, International Classification of Diseases, tenth edition.

## Algorithms

We devised strategies for integrating and reconciling multiple sources of information, where available, for key variables. These algorithms were implemented in R during data processing, but where the sources of information were complex, diagrams were developed to aid explanation and discussion with clinical colleagues [1,2] (Morley 2014; Denaxas & Morley 2015). Table [S2](#_bookmark7) shows which data sources were used in the derivation of each variable in the data set.

**Table S2.** Data sources used included in variable definition.

| Variable | EPR | PSP | D | CDAA | UDS | AUDIT | RA | E | MHA | NLP |
| --- | --- | --- | --- | --- | --- | --- | --- | --- | --- | --- |
| Age | ✓ |  |  |  |  |  |  |  |  |  |
| Gender | ✓ |  |  |  |  |  |  |  |  |  |
| Ethnicity | ✓ | ✓ |  |  |  |  |  | ✓ |  |  |
| Housing status | ✓ | ✓ |  |  |  |  | ✓ | ✓ |  |  |
| Alcohol use |  | ✓ |  | ✓ |  | ✓ |  | ✓ |  | ✓ |
| Cannabis use |  | ✓ |  | ✓ | ✓ |  |  | ✓ |  |  |
| Stimulant use |  | ✓ |  | ✓ | ✓ |  |  | ✓ |  |  |
| Opiate use |  | ✓ |  | ✓ | ✓ |  |  | ✓ |  |  |
| Sedative use |  | ✓ |  | ✓ | ✓ |  |  | ✓ |  |  |
| Synthetic cannabinoid use |  | ✓ |  | ✓ |  |  |  | ✓ |  |  |
| Party drug use |  | ✓ |  | ✓ |  |  |  | ✓ |  |  |
| Psychiatric diagnosis |  |  | ✓ |  |  |  |  |  |  | ✓ |
| Detention outcome |  | ✓ |  |  |  |  |  | ✓ | ✓ |  |
| Suicidal intent at entry |  | ✓ |  |  |  |  |  | ✓ |  |  |
| Suicidal intent at exit |  | ✓ |  |  |  |  |  | ✓ |  |  |

AUDIT, Alcohol Use Disorders Identification Test form; CDAA, Current Drug And Alcohol form; D, diagnosis; E, event notes; EPR, centralised electronic patient records; MHA, Mental Health Act records; NLP, natural language processing algorithm output; PSP, Place of Safety Proforma; RA, risk assessment form; UDS, urine drug screen;

For most phenotypes, integration of information from multiple sources was straightforward, with preference given to information originating from the PoS proforma. However, for some phenotypes, such as those relating to substance use, there was a range of information available which had to be reconciled and integrated. For these phenotypes, more complex algorithms were developed as detailed below.

### Acute alcohol use

For this phenotype, the aim was to identify acute alcohol use (i.e. in the previous 24-48 hours leading up to detention), as opposed to long-term dependence. Consequently, we gave priority to information that provided immediate, biological indications of the effects of alcohol: blood alcohol concentration and/or alcohol withdrawal assessment, as recorded in clinical notes, and administration of chlordiazepoxide (medication used in the treatment of alcohol withdrawal), which was drawn from NLP output (see Figure [S2).](#_bookmark9) We then included information from two structured forms – the PoS proforma record of alcohol intoxication, and the Current Drug and Alcohol form which records substances used on the day of detention and the day prior. Finally, we incorporated information from clinical notes on self-reported alcohol use, and the assessments of police, paramedics, and clinical staff. If there was no information regarding alcohol use, but there was a record of using other substances, then we classified this as no evidence of alcohol use. However, if there was no information about any substance use for a particular detention, acute alcohol use was classified as missing.


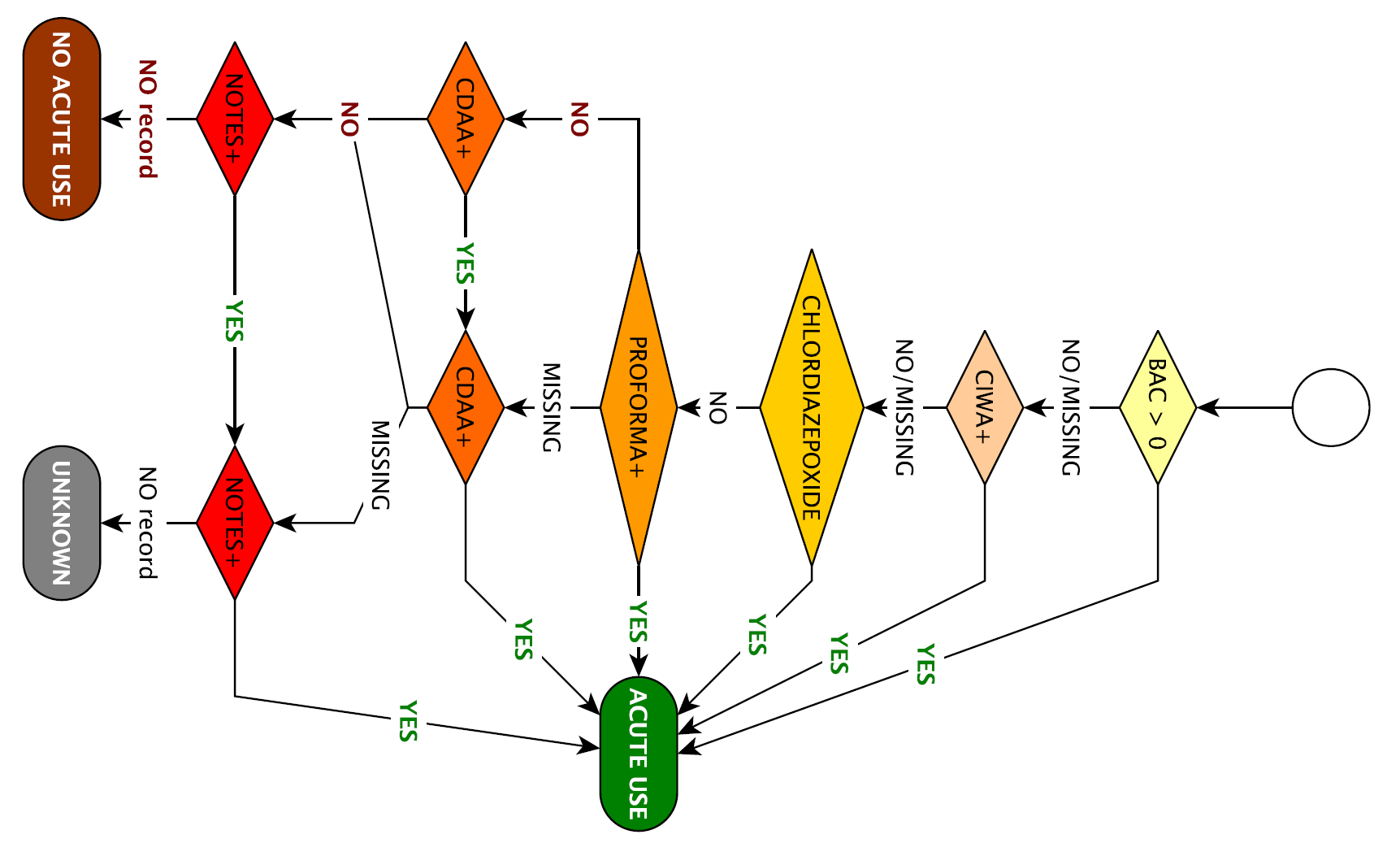


**Figure S2.** Diagram of algorithm for integrating sources of information on acute alcohol use. BAC, blood alcohol concentration; CDAA+, record of acute alcohol use in Current Drug And Alcohol form; CIWA, Clinical Institute Withdrawal Assessment of Alcohol score; NOTES+, record of acute alcohol use from clinical note scoring; PROFORMA+, record of alcohol intoxication in PoS proforma.

### Recent illicit and licit drug use

For all substances of interest, apart from alcohol, the same sources of information, or a subset, were available and thus a single algorithm could be used. As for alcohol use, we prioritised biological measures of recent use, so first examined data from urine drug screens where available. Following that, we prioritised the same sources of structured information and data extracted from clinical notes as the alcohol use algorithm (see Figure [S3).](#_bookmark11) The algorithm was applied to create variables indicating recent use of cannabis, stimulants (amphetamines, cocaine), opiates (heroin, methadone, buprenorphine), sedatives, synthetic cannabinoids, party drugs (MDMA, G, GHB/GBH).


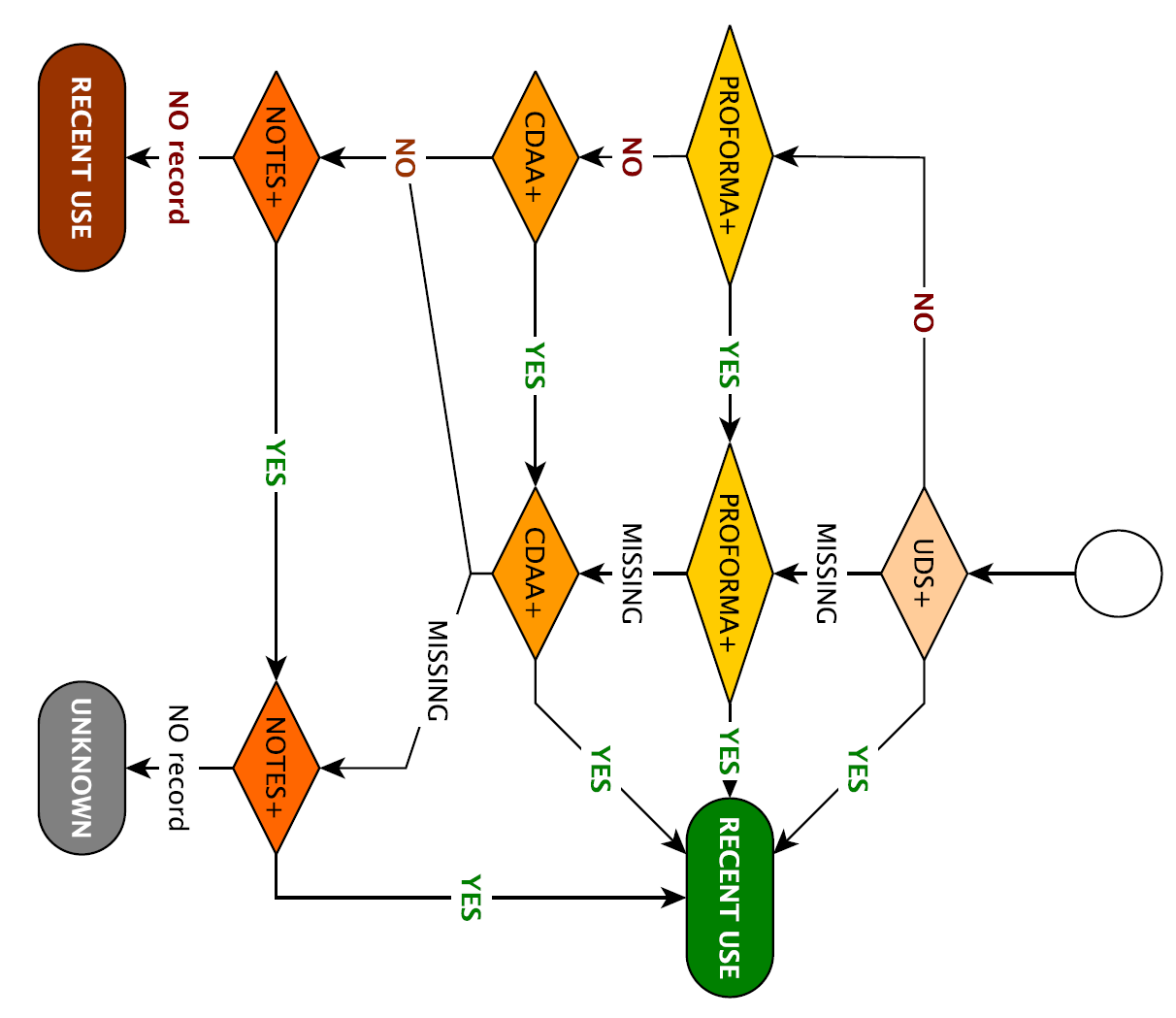


**Figure S3.** Diagram of algorithm for integrating sources of information on recent substance use. CDAA+, record of recent use of drug in Current Drug And Alcohol form; NOTES+, record of recent use of drug from clinical note scoring; PROFORMA+: record of intoxication due to substance in PoS proforma; UDS, urine drug screen.

### Detention outcome and destination

Detention outcome was ultimately classified as: *Admission – Informal*, *Admission – Section 2/3*, *Discharge – Mental Health follow-up* or *Discharge – GP follow-up*. The destinations included: *inpatient care*, *GP*, *Community Mental Health Team* or *Home Treatment Team*. Consequently, information about one variable could be used to infer the value of the other variable if it was missing. We initially used structured information from the PoS proforma to derive these measures. Where this information was missing, we incorporated information from the Events table, and finally from manual scoring of clinical notes. We cross-checked this information against records in the *Mental Health Act* table to determine whether admissions were informal or under Section 2 or Section 3 of the MHA. If we could not determine the conditions under which someone was admitted, this was treated as missing.

### Past year psychiatric diagnoses

Information on psychiatric diagnoses recorded during the past year (up to and including the current detention episode) were drawn from the structured diagnosis table and from the output of a previously developed natural language processing algorithm [3] (Perera 2016). Table [S3](#_bookmark14) displays ICD-10 codes included and the categorisation used for the phenotype algorithm. Where multiple diagnoses existed that belonged to different categories, diagnoses relating to psychotic illnesses were retained in preference to those relating to non-psychotic illnesses. Individuals with a diagnosis in the learning disorder and/or neurodevelopmental, or organic disorders categories, were excluded from the data set (see main text).

**Table S3.** ICD-10 codes included in past-year psychiatric diagnoses phenotype. LD indicates learning disability.

| **Code** | **Description** | **Categorisation** |
| --- | --- | --- |
| F02.* | Dementia in other diseases classified elsewhere | Organic disorders |
| F03 | Unspecified dementia | Organic disorders |
| F05.* | Delirium, not induced by alcohol and other psychoactive substances | Organic disorders |
| F06.* | Other mental disorders due to brain damage and dysfunction and to physical disease | Organic disorders |
| F07.* | Personality and behavioural disorders due to brain disease, damage  and dysfunction | Organic disorders |
| F09.* | Unspecified organic or symptomatic mental disorder | Organic disorders |
| F20.* | Schizophrenia | Psychotic illness |
| F21.* | Schizotypal disorder | Psychotic illness |
| F22.* | Persistent delusional disorders | Psychotic illness |
| F23.* | Acute and transient psychotic disorders | Psychotic illness |
| F25.* | Schizoaffective disorders | Psychotic illness |
| F28 | Other nonorganic psychotic disorders | Psychotic illness |
| F29 | Unspecified nonorganic psychosis | Psychotic illness |
| F30.* | Manic episode | Psychotic illness |
| F31.* | Bipolar affective disorder | Psychotic illness |
| F32.* | Depressive episode | Non-psychotic illness |
| F33.* | Recurrent depressive disorder | Non-psychotic illness |
| F34.* | Persistent mood [affective] disorders | Non-psychotic illness |
| F38.* | Other mood [affective] disorders | Non-psychotic illness |
| F39 | Unspecified mood [affective] disorder | Non-psychotic illness |
| F40.* | Phobic anxiety disorders | Non-psychotic illness |
| F41.* | Other anxiety disorders | Non-psychotic illness |
| F42.* | Obsessive-compulsive disorder | Non-psychotic illness |
| F43.* | Reaction to severe stress, and adjustment disorders | Non-psychotic illness |
| F44.* | Dissociative [conversion] disorders | Non-psychotic illness |
| F45.* | Somatoform disorders | Non-psychotic illness |
| F50.* | Eating disorders | Non-psychotic illness |
| F52.* | Sexual dysfunction, not caused by organic disorder or disease | Non-psychotic illness |
| F54.* | Psychological and behavioural factors associated with disorders or diseases classified elsewhere | Non-psychotic illness |
| F60.* | Specific personality disorders | Non-psychotic illness |
| F61 | Mixed and other personality disorders | Non-psychotic illness |
| F62.* | Enduring personality changes, not attributable to brain damage and disease | Non-psychotic illness |
| F63.* | Habit and impulse disorders | Non-psychotic illness |
| F68.* | Other disorders of adult personality and behaviour | Non-psychotic illness |
| F69 | Unspecified disorder of adult personality and behaviour | Non-psychotic illness |
| F70 | Mild mental retardation | LD/neurodevelopmental |
| F71 | Moderate mental retardation | LD/neurodevelopmental |
| F72 | Severe mental retardation | LD/neurodevelopmental |
| F78 | Other mental retardation | LD/neurodevelopmental |
| F79 | Unspecified mental retardation | LD/neurodevelopmental |
| F80.* | Specific developmental disorders of speech and language | LD/neurodevelopmental |
| F81.* | Specific developmental disorders of scholastic skills | LD/neurodevelopmental |
| F82 | Specific developmental disorder of motor function | LD/neurodevelopmental |
| F84.* | Pervasive developmental disorders | LD/neurodevelopmental |
| F88 | Other disorders of psychological development | LD/neurodevelopmental |
| F89 | Unspecified disorder of psychological development | LD/neurodevelopmental |
| F44.* | Dissociative [conversion] disorders | Non-psychotic illness |
| F45.* | Somatoform disorders | Non-psychotic illness |
| F50.* | Eating disorders | Non-psychotic illness |
| F52.* | Sexual dysfunction, not caused by organic disorder or disease | Non-psychotic illness |
| F54.* | Psychological and behavioural factors associated with disorders or diseases classified elsewhere | Non-psychotic illness |
| F60.* | Specific personality disorders | Non-psychotic illness |
| F61 | Mixed and other personality disorders | Non-psychotic illness |
| F62.* | Enduring personality changes, not attributable to brain damage and disease | Non-psychotic illness |
| F63.* | Habit and impulse disorders | Non-psychotic illness |
| F68.* | Other disorders of adult personality and behaviour | Non-psychotic illness |
| F69 | Unspecified disorder of adult personality and behaviour | Non-psychotic illness |
| F70 | Mild mental retardation | LD/neurodevelopmental |
| F71 | Moderate mental retardation | LD/neurodevelopmental |
| F72 | Severe mental retardation | LD/neurodevelopmental |
| F78 | Other mental retardation | LD/neurodevelopmental |
| F79 | Unspecified mental retardation | LD/neurodevelopmental |
| F80.* | Specific developmental disorders of speech and language | LD/neurodevelopmental |
| F81.* | Specific developmental disorders of scholastic skills | LD/neurodevelopmental |
| F82 | Specific developmental disorder of motor function | LD/neurodevelopmental |
| F84.* | Pervasive developmental disorders | LD/neurodevelopmental |
| F88 | Other disorders of psychological development | LD/neurodevelopmental |
| F89 | Unspecified disorder of psychological development | LD/neurodevelopmental |
| F90.* | Hyperkinetic disorders | LD/neurodevelopmental |
| F91.* | Conduct disorders | LD/neurodevelopmental |
| F93.* | Emotional disorders with onset specific to childhood | LD/neurodevelopmental |
| F94.* | Disorders of social functioning with onset specific to childhood and adolescence | LD/neurodevelopmental |
| F95.* | Tic disorders | LD/neurodevelopmental |
| F98.* | Other behavioural and emotional disorders with onset usually occurring in childhood and adolescence | LD/neurodevelopmental |
| F99 | Mental disorder, not otherwise specified | Non-psychotic illness |
| G10 | Huntington’s disease | Organic disorders |
| Q90 | Down’s syndrome | LD/neurodevelopmental |

ICD-10, International Classification of Diseases, Tenth Revision.

# Multiple Imputation

## Approach

Imputation was conducted using Multiple Imputation by Chained Equations (MICE) as implemented in the MICE package for the R software [4] (van Buuren 2011) following previously published guidance for implementation and reporting [5-7] (White 2011, Sterne 2009, Hayati Rezvan 2015). We initially investigated variable-wise and participant-wise missing data, and the distribution of variables in complete cases versus those with missing data. As recommended, the multiple imputation model included the primary and secondary outcomes, all predictors, accounting for pre-planned interactions, and relevant auxiliary variables [8-11] (Kontopantelis 2017, Harel 2018, Perkins 2018, Tilling 2016); details provided in Table [S4.](#_bookmark17) A total of 50 imputed data sets were generated and results from analyses of the individual datasets combined using Rubin’s rules [12] (Rubin 1987).

**Table S4.** Variables included in multiple imputation model

| **Type** | **Description** |
| --- | --- |
| Outcomes | Detention outcome |
|  | Destination after detention |
| Predictors | Age and gender |
|  | Housing status |
|  | Ethnicity |
|  | Recent use of other drugs |
|  | Past-year psychiatric diagnosis |
|  | Acute alcohol use |
|  | Suicidal thoughts at end of detention |
|  | Interaction between alcohol use and suicidal thoughts |
| Auxiliary | Presence of PoS proforma |
|  | Suicide method |
|  | Indicator of first or subsequent detention |

We carried out a diagnostic check of the imputation model by comparing variable distributions from observed and imputed data sets, and compared results of the primary analysis using multiply imputed data to those from a complete record analysis. We also conducted sensitivity analyses to assess the impact of departures from the Missing At Random assumption. As Hayati Rezvan *et al.* [7] (2015) note, there are multiple approaches to testing this assumption. van Buuren (2018) [13] discusses using a *δ*-adjustment to explore the impact of variation in the missing data, had they been observed; following this we modelled a range of scenarios for the ‘true’ values of the missing data for our two main predictors – recent alcohol use and suicidal thoughts at end of detention. We generated imputations as described above (same model and number of data sets) under four different scenarios, one for each possible value that the missing data for each variable could take. van Buuren (2018) [13] notes these types of scenarios are extreme and sensitivity analyses should preferably be based on realistic scenarios. However, as we had no prior hypotheses for why data may be missing, sensitivity analysis allowed us to explore how different scenarios may affect the robustness of our results.

## Results

We found little variation in the distribution of variables between the observed and imputed data sets. There was only slight variation in the point estimates for the regression coefficients between the imputed data and the observed data, which was expected given that data were only missing for a few variables, and the overall level of missing data was relatively low (see Table [S5](#_bookmark19) for a comparison of results from the imputed data set and complete record analysis, and Figure [S4](#_bookmark21) for graphical comparison).

**Table S5.** Comparison of results from the multivariable regression analysis using multiply imputed data or complete records. Data are shown as odds ratio (95% confidence interval); *P*-value.

|  | **Imputed (n=650)** | **Complete cases (n=483)** |
| --- | --- | --- |
| Diagnosis – none | 1.3 (0.8-2.2); 0.34 | 1.7 (0.9-3.3); 0.09 |
| Diagnosis – psychotic illness | 5.1 (3.1-8.4); <0.001 | 5.2 (2.9-9.4); <0.001 |
| Drug use | 1 (0.6-1.5); 0.9 | 0.9 (0.6-1.5); 0.75 |
| Ethnicity | 1.6 (1-2.5); 0.05 | 1.6 (0.9-2.7); 0.09 |
| Housing | 1.4 (0.9-2.4); 0.15 | 1.3 (0.7-2.2); 0.37 |
| Age | 1 (1-1); 0.11 | 1 (1-1); 0.02 |
| Gender | 1.1 (0.7-1.8); 0.55 | 1.4 (0.9-2.4); 0.17 |
| Alcohol use | 0.4 (0.2-0.6); <0.001 | 0.4 (0.2-0.6); <0.001 |
| Suicidality at end | 8.2 (3.5-19.1); <0.001 | 9.8 (3.8-25.3); <0.001 |
| Alcohol use + suicidality at end | 3.6 (1.8-7.1); <.001 | 4 (1.8-8.7); <0.001 |

The estimates from the regression analyses of the data sets imputed for the four different sensitivity analysis scenarios were relatively consistent (see Figure [S5](#_bookmark22) and Table [S6).](#_bookmark20) The confidence intervals for the estimates across the scenarios overlapped and the point estimates were very similar. Given that these scenarios represent extremes, the results suggest that overall the analyses are robust.

**Table S6.** Comparison of regression estimates from multiple imputation sensitivity analyses. Estimates shown as regression coefficients (95% confidence interval). *AAU_m_*: indicates value of missing data for acute alcohol use; *SDE_m_*: indicates value of missing data for suicidal thoughts at detention end

| Coefficient | Main | AAUm = ‘Yes’ | AAUm = ‘No’ | SDEm = ‘No’ | SDEm = ‘Yes’ |
| --- | --- | --- | --- | --- | --- |
| Alcohol use | 0.9 (0.5-1.3) | 0.9 (0.5-1.4) | 0.9 (0.5-1.4) | 0.9 (0.5-1.4) | 0.9 (0.5-1.4) |
| Suicidal at end | 2 (1.4-2.5) | 1.9 (1.4-2.4) | 1.9 (1.4-2.4) | 1.9 (1.4-2.4) | 1.9 (1.4-2.4) |
| Alcohol use – suicidal at end | 0.3 (-0.5-1.1) | 0.3 (-0.5-1.1) | 0.3 (-0.5-1.1) | 0.3 (-0.5-1.1) | 0.3 (-0.5-1.1) |
| Diagnosis – none | 0.1 (-0.4-0.6) | 0.1 (-0.4-0.6) | 0.1 (-0.4-0.6) | 0.1 (-0.4-0.6) | 0.1 (-0.4-0.6) |
| Diagnosis – psychotic illness | 1.4 (1-1.8) | 1.3 (0.9-1.8) | 1.3 (0.9-1.8) | 1.3 (0.9-1.8) | 1.3 (0.9-1.8) |
| Other drug use | 0 (-0.4-0.4) | 0.1 (-0.3-0.4) | 0.1 (-0.3-0.4) | 0.1 (-0.3-0.4) | 0.1 (-0.3-0.4) |
| Ethnicity | 0.5 (0.1-0.9) | 0.5 (0.1-0.9) | 0.5 (0.1-0.9) | 0.5 (0.1-0.9) | 0.5 (0.1-0.9) |
| Housing | 0.2 (-0.2-0.6) | 0.2 (-0.2-0.7) | 0.2 (-0.2-0.7) | 0.2 (-0.2-0.7) | 0.2 (-0.2-0.7) |
| Age | 0.01 (0-0) | 0.01 (0-0) | 0.01 (0-0) | 0.01 (0-0) | 0.01 (0-0) |
| Gender | 0.2 (-0.2-0.6) | 0.1 (-0.3-0.5) | 0.1 (-0.3-0.5) | 0.1 (-0.3-0.5) | 0.1 (-0.3-0.5) |

Alcohol use + Suicidality at end

●

●

●

●

●

●

●

●

●

●

●

●

●

●

●

●

●

●

●

●

Suicidality at end

Alcohol use

Gender

Age

Coefficient

Data

Housing

~~●~~ First detentions − imputed

~~●~~ First detentions − complete cases

Ethnicity

Drug use

Diagnosis − psychotic illness

Diagnosis − none

0.2 0.5 1.0 2.0 5.0 10.0 20.0

Estimate

**Figure S4.** Comparison of estimates (odds ratios with 95% confidence intervals) from the imputed and complete record analyses examining associations with primary outcome of detention outcome.

No alcohol + suicidal at end

●

●

●

●

●

●

●

●

●

●

●

●

●

●

●

Data

Suicidal at end

Coefficient

- Main
- Alcohol = No
- Alcohol = Yes
- Suicidal at end = No
- Suicidal at end = Yes

No alcohol

1 2 5 10 20 40

Estimate

**Figure S5.** Comparison of estimates (odds ratios with 95% confidence intervals) from the main multiple imputation analysis and sensitivity analyses under the different scenarios for missing data values.

# Sensitivity Analyses

## Approach

We conducted the main analyses using the first detention from each individual, even though some individuals appeared in our data set more than once as they were detained multiple times during the study period. To assess the impact of this on results, we re-analysed our data using data from all the detentions of each individual. As this is only a sensitivity analysis, we used a simple approach that did not account for the clustering of detentions within individuals. We conducted this sensitivity analysis using both the multiply imputed data and complete records.

## Results

As shown in Table [S7](#_bookmark26) and Figure [S6,](#_bookmark27) overall the results from the sensitivity analysis were very similar to those from the full data set.

**Table S7.** Comparison of interaction results from the sensitivity analysis using multiply imputed data or complete records. Estimates are displayed as odds ratios (95% confidence interval); *P*-value.

| **Acute alcohol use** | **Suicidal at detention end** | |
| --- | --- | --- |
|  | **No** | **Yes** |
| *Imputed* |  |  |
| No | ref. | 10.9 (5.5-21.8); *<*0*.*001 |
| Yes | 0.4 (0.27-0.69); *<*0*.*001 | 2.9 (1.7-4.9); *<*0*.*001 |
| *Complete* |  |  |
| No | ref. | 9.2 (4.9-17.3); *<*0*.*001 |
| Yes | 0.4 (0.26-0.63); *<*0*.*001 | 2.9 (1.7-4.9); *<*0*.*001 |

Alcohol use + Suicidality at end

●

●

●

●

●

●

●

●

●

●

●

●

●

●

●

●

●

●

●

●

●

●

●

●

●

●

●

●

●

●

●

●

●

●

●

●

●

●

●

●

Suicidality at end

Alcohol use

Gender

Data

Age

Coefficient

Housing

Ethnicity

~~●~~ First detentions − imputed

~~●~~ First detentions − complete cases

~~●~~ All detentions − imputed

~~●~~ All detentions − complete cases

Drug use

Diagnosis − psychotic illness

Diagnosis − none

0.2 0.5 1.0 2.0 5.0 10.0 20.0

Estimate

**Figure S6:** Comparison of estimates (odds ratios with 95% confidence intervals) from the four analyses examining associations with primary outcome: imputed first detentions, complete case first detentions, imputed all detentions, complete case all detentions.

# References

1. Morley KI, Wallace J, Denaxas SC *et al*. Defining disease phenotypes using national linked electronic health records: A case study of atrial fibrillation. PLoS One 2014;9:e110900.
2. Denaxas SC, Morley KI. Big biomedical data and cardiovascular disease research: Opportunities and challenges. Eur Heart J Qual Care Clin Outcomes 2015;1:9-16.
3. Perera G, Broadbent M, Callard F *et al*. Cohort profile of the South London and Maudsley NHS Foundation Trust Biomedical Research Centre (SLaM BRC) Case Register: current status and recent enhancement of an Electronic Mental Health Record-derived data resource. BMJ Open 2016;6:e008721.
4. van Buuren S, Groothuis-Oudshoorn K. Multivariate imputation by chained equations in R. J Statist Softw 2011;45:1-67.
5. White IR, Royston P, Wood AM. Multiple imputation using chained equations: Issues and guidance for practice. Stat Med 2011;30:377-99.
6. Sterne JAC, White IR, Carlin JB et al. Multiple imputation for missing data in epidemiological and clinical research: potential and pitfalls. BMJ 2009;338:b2393.
7. Hayati Rezvan P, Lee KJ, Simpson JA. The rise of multiple imputation: A review of the reporting and implementation of the method in medical research. BMC Med Res Methodol 2015;15:30.
8. Kontopantelis E, White IR, Sperrin M, Buchan I. Outcome-sensitive multiple imputation: A simulation study. BMC Med Res Methodol 2017;17:2.
9. Harel O, Mitchell EM, Perkins NJ et al. Multiple imputation for incomplete data in epidemiologic studies. Am J Epidemiol 2018;187:576-84.
10. Perkins NJ, Cole SR, Harel O et al. Principled approaches to missing data in epidemiologic studies. Am J Epidemiol 2018;187:568-75.
11. Tilling K, Williamson EJ, Spratt M, Sterne JAC, Carpenter JR. Appropriate inclusion of interactions was needed to avoid bias in multiple imputation. J Clin Epidemiol 2016;80:107-15.
12. Rubin DB. Multiple Imputation for Nonresponse in Surveys. New York: John Wiley & Sons Inc.; 1987.
13. van Buuren S. Flexible Imputation of Missing Data. 2nd ed. FL: Boca Raton: CRC/Chapman & Hall; 2018.
